# A Sesquiterpenoid from Farfarae Flos Induces Apoptosis of MDA-MB-231 Human Breast Cancer Cells through Inhibition of JAK–STAT3 Signaling

**DOI:** 10.3390/biom9070278

**Published:** 2019-07-13

**Authors:** Hyeri Jang, Hyejin Ko, Kwangho Song, Yeong Shik Kim

**Affiliations:** Natural Products Research Institute, College of Pharmacy, Seoul National University, 1 Gwanak-ro, Gwanak-gu, Seoul 08826, Korea

**Keywords:** Farfarae Flos, sesquiterpenoid, anticancer, STAT3, triple-negative breast cancer (TNBC), MDA-MB-231 cells

## Abstract

Triple-negative breast cancers (TNBCs) are hard-to-treat breast tumors with poor prognosis, which need to be treated by chemotherapy. Signal transducer and activator of transcription 3 (STAT3) is a transcription factor involved in proliferation, metastasis, and invasion of cancer cells. Therefore, research on searching for promising compounds with metabolism that suppress phosphorylation or transcription of STAT3 in TNBC cells is important. Farfarae Flos is well known as a traditional medicine for treating inflammation. However, few studies have shown that sesquiterpenoids from Farfarae Flos have an anticancer effect. In this study, efficient separation methods and an MTT assay were conducted to isolate an anticancer compound from Farfarae Flos against TNBC MDA-MB-231 cells. Here, 7β-(3-Ethyl-*cis*-crotonoyloxy)-1α-(2-methylbutyryloxy)-3,14-dehydro-*Z*-notonipetranone (ECN), a compound isolated from Farfarae Flos showed a potent cytotoxic effect on MDA-MB-231 cells. ECN inhibited JAK–STAT3 signaling and suppressed the expression of STAT3 target genes. In addition, ECN induced apoptosis through both extrinsic and intrinsic pathways. Furthermore, we investigated that ECN inhibited the growth of tumors by intraperitoneal administration in mice injected with MDA-MB-231 cells. Therefore, ECN can be an effective chemotherapeutic agent for breast cancer treatment.

## 1. Introduction

Breast cancer is the most common cancer and the leading cause of cancer-related death among women [1]. Researchers have identified hormonal, lifestyle, and environmental factors that may increase the risk of breast cancer [2]. However, it is not clear how to define the causes of developing cancer [3,4]. Although further improvements in cancer biology have allowed the development of systemic treatments, hormonal therapies, and target drugs, better understanding of breast cancer is needed to refine disease mechanisms and molecular characteristics to improve drug development and treatment approaches [5]. Breast cancer is a heterogeneous disease. Gene expression analysis identified breast cancer subtypes, including basal-like, human epidermal growth factor receptor type 2 (HER2) positive/estrogen receptor negative, luminal A, and luminal B [6]. Triple-negative breast cancer (TNBC) is defined as tumors that lack expression of an estrogen receptor, progesterone receptor, and HER2 [7,8,9]. It appears in 12% to 17% of women with breast cancer who cannot be treated with endocrine therapy or therapies targeted to HER2 [10]. In addition, the mortality rates appear to be increased for 5 years after diagnosis [11]. Hence, it needs to have more studies of TNBC and treatment for prevention of incidence and death.

The signal transducers and activators of transcription (STAT) proteins were identified as transcription factors that were critical in mediating virtually all cytokine-driven signaling [12]. These proteins are latent in the cytoplasm and become activated through tyrosine phosphorylation which typically occurs through cytokines, Janus kinase (JAKs), and growth factor receptor tyrosine kinases [12,13,14]. Phosphorylated STATs form homo- or heterodimers, enter the nucleus, and working coordinatively with other transcription factors, lead to increased transcriptional initiation [15,16,17]. The STAT proteins, especially STAT1, 3, and 5, are persistently tyrosine phosphorylated and activated, and play an important role in controlling cell cycle progression and apoptosis [18,19,20,21]. STAT3, a member of the STAT proteins, is a crucial mediator of oncogenic signaling [22]. It is involved in a variety of biological functions, including cell growth, suppression and induction of apoptosis, and cell motility [23,24,25]. The constitutive activation of STAT3 is frequently detected in breast carcinoma cell lines [26]. It has been reported that breast tumors contain activated STAT3 [27]. In addition, studies have shown that the JAKs phosphorylate STAT3 in response to stimulation by cytokines or growth factor [28,29]. Therefore, JAK–STAT3 is a novel molecular target signaling for the development of breast cancer therapeutics [30]. 

Farfarae Flos is a perennial medicinal plant that belongs to the Asteraceae family. It is distributed in Korea, China, Asia, North Africa, and Europe. Dried flower buds of *Tussilago farfara* (Farfarae Flos) are well known as traditional medicine for treating severe cough, bronchitis, and asthma [31]. In addition, it is reported that Farfarae Flos contains sesquiterpenoids, terpenoids, steroids, and flavonoids [32,33,34,35]. In particular, sesquiterpenoids showed anti-inflammation, antioxidant, and neuroprotective activities [36,37,38]. In this study, we isolated a sesquiterpenoid from Farfarae Flos and examined its inhibitory effect on STAT3 activation inducing apoptosis in MDA-MB-231 human breast cancer cells both in vitro and in vivo.

## 2. Materials and Methods 

### 2.1. Apparatus

To isolate compounds, countercurrent chromatography (CCC) was carried out with a model TBE-1000A (Tauto Biotech., Shanghai, China), including a six-port injection valve, a 60 mL sample loop, a Hitachi L-6200 pump, and a Hitachi UV detector L-7400 (Hitachi, Tokyo, Japan). The CCC had three multilayer coil separation columns connected in series (3.0 mm inside diameter (ID) of tube, each volume of three coils: 330 mL, total volume: 1000 mL). The rotation speed of the apparatus ranged from 0 to 500 rpm. High-performance liquid chromatography (HPLC) analyses were carried out on a Hitachi L-6200 instrument equipped with a Hitachi L-4000 UV detector (Hitachi, Tokyo, Japan) and a SIL-9A auto injector (Shimadzu, Japan). A YMC-Triart C_18_ column for analyzing samples (4.6 × 150 mm ID, 5 μm particle size) was purchased from YMC Co. Ltd. (Seongnam, Korea). The preparative-HPLC separation was performed using a Hitachi JP/L-7100 equipped with a Hitachi L-4000 UV detector (Hitachi, Tokyo, Japan). A YMC-Triart C_18_ column for preparative-HPLC (100 × 250 mm ID, 10 μm particle size) was purchased from YMC Co. Ltd. (Seongnam, Korea). The mass spectrometry (MS) analyses were performed using a Finnigan LCQ ion trap mass spectrometer from Thermo Finnigan (San Jose, CA, USA) equipped with an electrospray ionization (ESI) source. The nuclear magnetic resonance (NMR) analyses were recorded on Bruker Advance 600 spectrometers (Bruker, Rheinstetten, Germany). 

### 2.2. Reagents

Chemical reagents used for extraction, CCC, and column chromatography were of analytical grade. Acetonitrile (ACN), distilled water (DW), and methanol (MeOH), were purchased from DaeJung Science (Seoul, Korea). The HPLC-grade acetonitrile was supplied by J.T. Baker (Phillipsburg, NJ, USA). Distilled water (NANO pure Diamond, Barnstead, NH, USA) was used for all solutions and dilutions. For cell culture, Dulbecco’s modified Eagles’ medium (DMEM), fetal bovine serum (FBS), penicillin-streptomycin solution, and HEPES buffer solution were purchased from GenDepot (Baker, TX, USA). Dulbecco’s phosphate buffered saline (DPBS), 3-(4,5-dimethylthiazol-2-yl)-2,5-diphenyltetrazolium bromide (MTT), and protease inhibitor cocktail were obtained from Sigma-Aldrich Co. (St. Louis, MO). Dimethyl sulfoxide (DMSO) (purity: >99.9%) and staurosporine for dissolving certain substrates for the cell culture were purchased from Sigma-Aldrich Co. (St. Louis, MO, USA). DMSO (purity: >99%) for the MTT assay was obtained from Duksan Pharmaceutical Co. (Ansan, Korea). 

The primary antibodies for procaspase 3, procaspase 8, STAT3, β-actin, epidermal growth factor receptor (EGFR), COX-2, and goat secondary antibodies were from Santa Cruz Biotechnology (Santa Cruz, CA, USA). The primary antibodies for cleaved caspase 3, p-STAT3 (Tyr705), p-EGRF, p-JAK1, and JAK2 were from Abcam (Cambridge, MA, USA).The primary antibodies for p-STAT3 (Ser727), cleaved caspase 8, p-JAK2, p-Src, Src, and Bcl-2 were from Cell Signaling Technology (Beverly, MA, USA). The primary antibodies for poly-(ADP-ribose) polymerase (PARP), JAK1, Src homology 2 domain-containing protein tyrosine phosphatase (SHP1), SHP2, Cyclin D1, proliferating cell nuclear antigen (PCNA), rabbit, and mouse secondary antibodies were from Gene Tex (Irvine, CA, USA). All other chemicals and reagents were purchased from Sigma-Aldrich Co. (St. Louis, MO, USA).

### 2.3. Plant Extract 

Farfarae Flos was purchased from Omniherb (Yeongcheon, Korea). Dried and chopped Farfarae Flos (2.4 kg) was extracted with 55% acetonitrile (3 × 5 L) by ultrasonication at room temperature for 12 h. 

### 2.4. Sesquiterpenoids Fraction of Farfarae Flos Using Countercurrent Chromatography (CCC)

CCC was filled with hexane as the upper phase (the stationary phase). The extract of Farfarae Flos as the lower phase (the mobile phase) was pumped into the system at a flow rate of 15 mL/min while the columns were rotating at a speed of 455 rpm [39]. The UV detection system was performed at 235 nm and 2.5 absorbance units. After the extract was injected, 55% acetonitrile was pumped into the system at the same flow rate. When the UV signal decreased, 100% acetonitrile was pumped into the column. The sesquiterpenoids fraction was collected according to the elution profile and analyzed by HPLC-UV. The fraction was evaporated under reduced pressure.

### 2.5. Isolation of Compounds Via Preparative-HPLC

A YMC-Triart C_18_ column (100 × 250 mm ID, 10 μm particle size) was used to isolate and purify thecompounds. The sesquiterpenoids fraction was dissolved in methanol and injected into the preparative-HPLC to separate 18 fractions (P1–P18-3). The preparative-HPLC condition for fractions was as follows: distilled water (A) and acetonitrile (B), gradient: 0–35 min (60–95% B); 35–36 min (95–100% B) and then washed with 100% B for 4 min at a flow rate of 4 mL/min. UV detection was conducted at 235 nm. 

### 2.6. HPLC Analysis

A YMC Triart C_18_ column (4.6 × 150 mm ID, 5 μm particle size) was used to analyze the extract, sesquiterpenoids fraction (CCC), sub-fractions (preparative-HPLC), and a compound at the last separation step. The injection volume was 20 μL. For analysis of each separation step, the mobile phase was optimized with distilled water (A) and acetonitrile (B). The gradient was the following system: 0–25 min (45–95% B); 25–27.5 min (95–100% B) and then equilibrated with 100% B for 5 min at a flow rate of 1.25 mL/min. The column was at room temperature and the UV detection was conducted at 220 nm. 

### 2.7. Identification of an Isolated Compound

An isolated compound was analyzed by ESI-MS (Thermo Finnigan LCQ ion trap mass spectrometer, Seoul National University, Seoul, Korea) for molecular weight. The compound was dissolved in CDCl_3_ and its structures was identified by comparing the ^1^H and ^13^C NMR (Bruker Advance 600-MHz, National Center for Interuniversity Research Facilities at Seoul National University, Seoul, Koreaspectra with references [33].

### 2.8. Cell Culture

MDA-MB-231 human breast cancer cells were obtained from the Korea Cell Bank (Seoul, Korea). MDA-MB-231 cells were maintained in Dulbecco’s modified Eagles’ medium (DMEM) supplemented with 10% fetal bovine serum (FBS) and antibiotics (penicillin 100 μg/mL and streptomycin 100 μg/mL) at 37 °C in 5% CO_2_ incubator.

### 2.9. Cell Viability Assay

Cell viability was measured by an MTT assay. The cells were seeded into 96-well plates at a density of 10^4^ cells/well and maintained at 37 °C in a humidified 5% CO_2_ incubator for 24 h. The compounds were added to each well and the cells were incubated for 0–48 h. The MTT solution (0.5 mg/mL) was added to each well and then incubated for 2 h. Following removal of media, the formazan crystals were dissolved in 100 μL of DMSO. Staurosporine was used as a positive control. The cell viability was assessed by measuring the absorbance at 595 nm wavelength using an Emax microplate reader (Molecular Devices, Sunnyvale, CA, USA). The relative cell viability was calculated and compared with the absorbance of the untreated control group. All experiments were performed in triplicate. 

### 2.10. Luciferase Reporter Assay

The cells were plated in 24-well plates at a density of 1×10^5^ cells/well and incubated at 37 °C in a humidified 5% CO_2_ incubator. After 24 h, the cells were transiently transfected with pstat3-Luc reporter vector in the presence of pCMV-Luc vector using a transfection reagent (Intron Biotechnology, Seoul, Korea). At 24 h post-transfection, the cells were treated with compounds for 24 h. A luciferase assay was performed using the dual luciferase reporter assay system (Promega, Madison, WI, USA). The luminescence signal was measured using a luminometer (MicroLumat Plus, Berthold Technologies, Dortmund, Germany).

### 2.11. Observation of Cell Morphology 

The cells were plated in 12-well plates at a density of 5 × 10^5^ cells/well and incubated at 37 °C in a humidified 5% CO_2_ incubator for 24 h. The cells were treated with the compounds for the indicated concentrations and cells were rewashed with DPBS. Cells were viewed with a CKX41 fluorescence microscope (Olympus, Tokyo, Japan) at a magnification of 200×. 

### 2.12. Annexin V/PI Staining

Apoptotic cells were differentiated from viable or necrotic cells using a combined staining of Annexin V and propidium iodide (PI). To examine the apoptosis-inducing potential of compounds, flow cytometry based Annexin V staining was performed to detect the externalization of phosphatidylserine. The cells were plated in 6-well plates at a density of 10^6^ cells/well and maintained at 37 °C in a humidified 5% CO_2_ incubator for 24 h. After treatment with compounds 24 h in different doses, adherent and floating cells were collected, washed with DPBS, and subsequently centrifuged. The cells were suspended in 1 × binding buffer and stained with Annexin V and PI according to the manufacturer’s directions (BD Biosciences, San Diego, CA, USA). After incubation for 15 min at room temperature in the dark, the cells were analyzed with flow cytometry using Becton Dickinson Fluorescence-activated cell sorting (FACS) Calibur (BD Biosciences, San Diego, CA, USA) to estimate the population of apoptotic cells.

### 2.13. Western Blot Analysis

The cells were seeded at a density of 10^6^ cells/well in 6-well plates and treated with the compounds for the indicated times and doses. After incubation, the adherent and floating cells were collected, washed with DPBS, and centrifuged. Whole cell lysates were prepared using a lysis buffer [20 mM HEPES (pH 7.6), 350 mM NaCl, 20% glycerol, 0.5 mM EDTA, 0.1 mM EGTA, 1% NP-40, 50 mM NaF, 0.1 mM DTT, 0.1 mM PMSF, and protease inhibitor cocktail] for 30 min on ice. The lysates were centrifuged at 15,000 rpm for 10 min. Nuclear extracts were prepared using a lysis buffer [10 mM HEPES (pH 7.9), 10 mM KCl, 0.1 mM EDTA, 0.1 mM EGTA, 1 mM DTT, 1 mM PMSF, and protease inhibitor cocktail] for 15 min on ice. Then, 10% NP-40 was added and the mixtures were centrifuged for 5 min. The nuclear pellets were resuspended in nuclear extraction buffer [20 mM HEPES (pH 7.9), 400 mM NaCl, 1 mM EDTA, 1 mM EGTA, 1 mM DTT, 1 mM PMSF, and protease inhibitor cocktail] and centrifuged at 15,000 rpm for 10 min. The tumor was homogenized and lysed in T-PER tissue protein extraction reagent (Thermo Fisher Scientific Inc., Rockford, IL, USA). The extract was centrifuged at 15,000 rpm for 10 min at 4 °C. 

The protein concentration was estimated using a Bradford reagent (Bio-Rad Laboratories Inc., Berkeley, CA, USA). An equal amount (20–30 μg) of protein was loaded on 8–12% SDS-polyacrylamide gels and transferred using a wet method to nitrocellulose membrane. After being blocked with 5% skim milk, the membrane was incubated at 4 °C overnight with specific primary antibodies. The membrane was washed and incubated at room temperature for 1 h with secondary antibodies conjugated with horseradish peroxidase. Finally, the blot was developed using a chemiluminescence kit (Ab Frontier, Seoul, Korea) and the immunoreactive band on the blot were visualized using a LAS-1000 image analyzer (Fuji Photo Film Co., Ltd., Tokyo, Japan). Densitometry was performed using ImageJ analysis software (Version 1.52, NIH, Bethesda, MD, USA).

### 2.14. Animals

Animal care experimental procedures were conducted in accordance with the procedures and guidelines approved by the Daegu-Gyeongbuk Medical Innovation Foundation Institutional Animal Care and Use committee [DGMIF-18010902-00]. Female BALB/c nude mice at the age of 7 weeks were purchased from the Central Animal Laboratory Inc. (Seoul, Korea).

### 2.15. Tumor Xenograft Study

MDA-MB-231 cells were subcutaneously injected into the right flanks of the mice. When the tumor volume reached around 100 mm^3^, mice were randomly divided into treatment and control groups (*n* = 10/group). The animals were intraperitoneally treated with ECN (1 mg/kg) every 2 days, whereas control animals were treated with an equal volume of DPBS. The body weight and tumor size were measured every 2 days. The tumor volume was determined by caliper measurements and calculated using the following formula: {length × (width)^2^}/2. After 21 days of treatment, the mice were sacrificed. The tumors were removed and weighed. Then, the tumors were immediately frozen in liquid nitrogen for further protein isolation.

### 2.16. Statistical Analysis

In vitro data were presented as the mean ± the standard deviation from three different experiments. In vivo data were presented as the mean ± the standard error of the mean. An analysis of variance (ANOVA) procedures were used for the statistical analysis of multiple comparisons. *P* values were considered statistically significant [* *P* < 0.05; ** *P* < 0.01; *** *P* < 0.001].

## 3. Results

### 3.1. Isolation and Identification of ECN 

**Figure 1:**
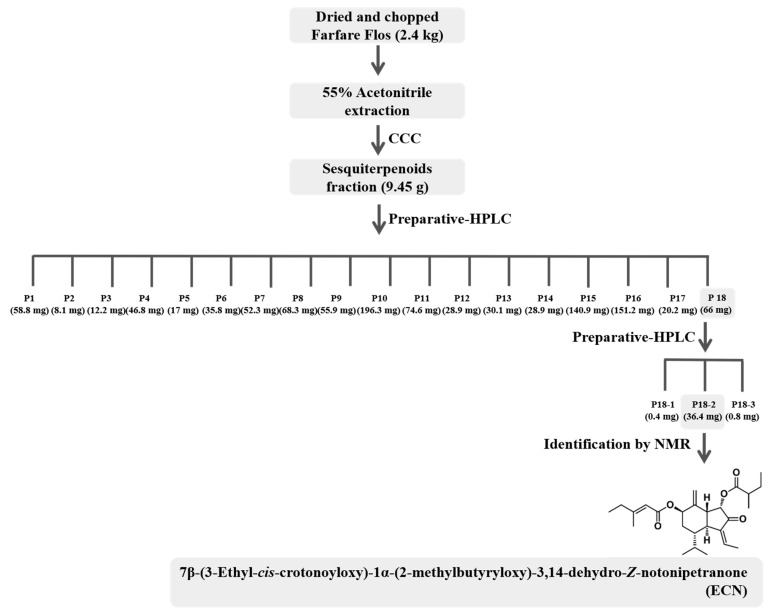
Outline of the separation of ECN. CCC: countercurrent chromatography.

**Figure 2:**
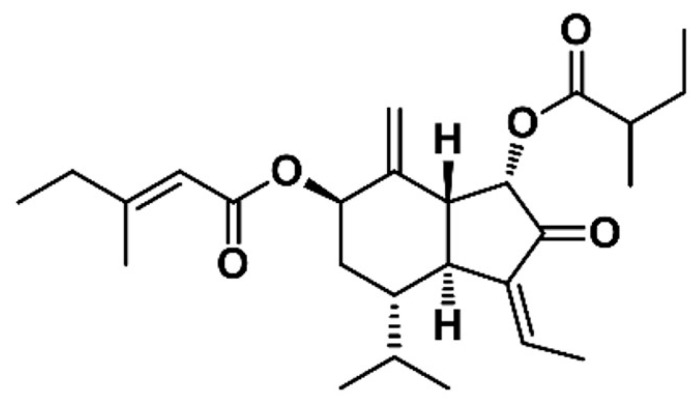
Chemical structure of ECN.

An anticancer compound from Farfarae Flos was isolated through activity-guided fractionation, as shown in Figure 1. This method is efficient for isolation of compounds with enrichment and purifying. The MTT assay was conducted with fractions obtained from preparative-HPLC to evaluate anticancer and cytotoxic effects on MDA-MB-231 cells. The fractions were tested with various concentrations and compared with a control, as shown in Table 1. The P18 fraction significantly suppressed cell proliferation. Hence, isolation using preparative-HPLC was performed to separate and purify the compound from the P18 fraction. The pure compound P18-2, obtained as a colorless oil, showed the most potent anticancer effect, as shown in Table 2. The chemical structure of the compound was determined based on the MS, ^1^H, and ^13^C NMR data. The [M + Na]^+^ ion at *m/z* 453.2622 (C_26_H_38_O_5_, Cal. 453.2617, error 1.1013 ppm) was detected in the ESI-MS spectrum, as shown in Figure S1. The ^1^H and ^13^C NMR spectra revealed that the presence of two esters, two olefinic, a ketone, a terminal methylene, and seven methyl signals, as shown in Table 3. Comparing NMR spectral data with the literature, the compound was identified as 7β-(3-ethyl-*cis*-crotonoyloxy)-1α-(2-methylbutyryloxy)-3,14-dehydro-*Z*-notonipetranone (ECN), as shown in Figure 2 [33].

### 3.2. Inhibitory Effect of ECN on STAT3 Activity in MDA-MB-231 Cells

**Figure 3:**
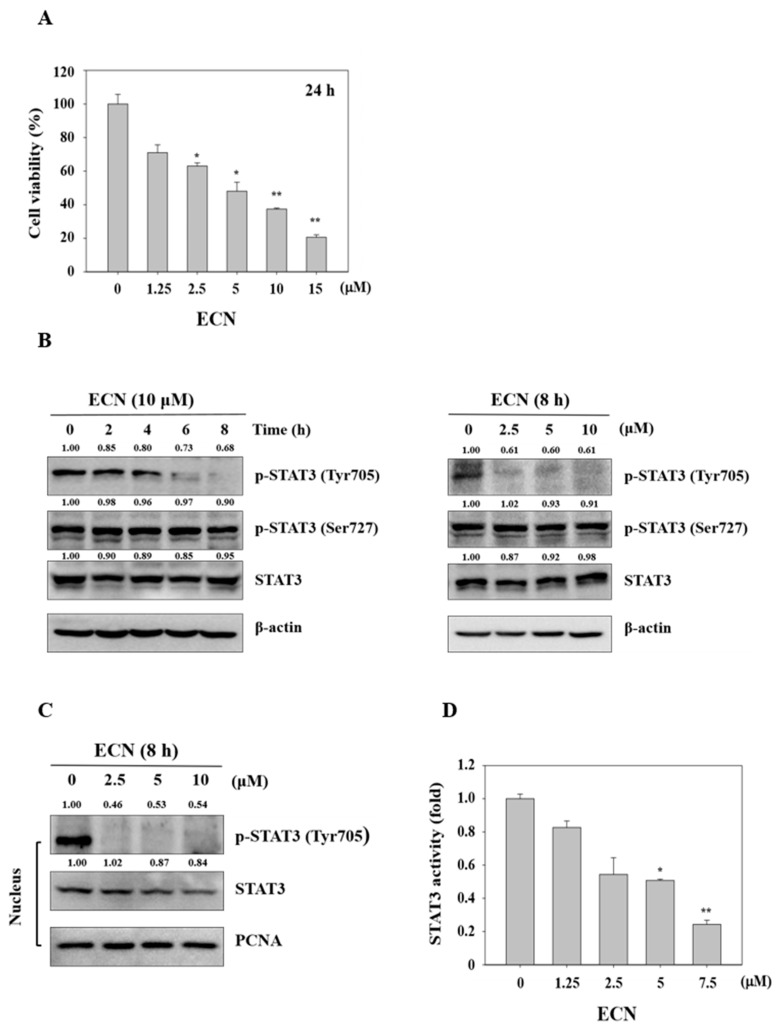
Cytotoxicity effect on ECN in MDA-MB-231 cells and the effect of ECN on STAT3 activation in MDA-MB-231 cells. (**A**) Cell viability was determined by 3-(4,5-dimethylthiazol-2-yl)-2,5-diphenyltetrazolium bromide (MTT) assay after 24 h ECN treatment. (**B**) The cells were treated with the indicated concentrations (right panel) and times (left panel) of ECN. The cell lysates were western blotted to determine p-STAT3 and STAT3 protein levels. (**C**) The nuclear extracts were western blotted to determine p-STAT3 and STAT3 protein levels. (**D**) Cells were transfected with the pstat3-Luc reporter vector and treated with ECN for 24 h. The luciferase assay was performed using the dual luciferase reporter assay system. Data were derived from three independent experiments and expressed as mean ± standard deviation. Significant difference compared with the control groups [* *P* < 0.05; ** *P* < 0.01].

MDA-MB-231 cells were exposed to various concentrations of ECN (0–15 μM) for 24 h and the cell viability was evaluated using the MTT assay. ECN significantly inhibited cell viability in a dose-dependent manner, as shown in Figure 3A. Next, we analyzed the effect of ECN on STAT3 activity in MDA-MB-231 cells by western blot analyses. The protein level of phosphorylation of STAT3 at serine and total STAT3 did not reduce. However, ECN significantly inhibited phosphorylation of STAT3 at tyrosine 705 in MDA-MB-231 cells in a dose and time-dependent manner, as shown in Figure 3B. Phosphorylation of tyrosine 705 promotes STAT3 dimerization and translocation to the nucleus, which is critical for STAT3 activation [40]. As shown in Figure 3C, ECN inhibited STAT3 translocation to the nucleus. These results suggested that ECN suppressed activation of STAT3 in cytoplasm and the nucleus of MDA-MB-231 cells. In addition, luciferase reporter gene assays were employed to detect STAT3 transcriptional activity. ECN suppressed STAT3 transcription activity in MDA-MB-231 cells, as shown in Figure 3D. 

### 3.3. Effect of ECN on STAT3 Upstream Signaling Pathways in MDA-MB-231 Cells

**Figure 4:**
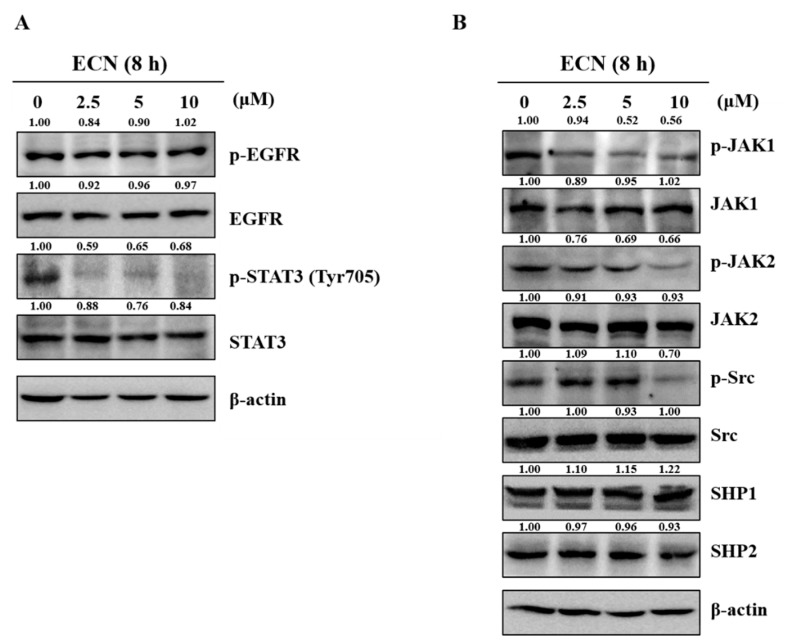
Effect of ECN on STAT3 upstream signaling in MDA-MB-231 cells. (**A**) The cells were treated with indicated concentrations of ECN for 8 h and the inhibitory effect of ECN on phosphorylation of epidermal growth factor receptor (EGFR) and STAT3 was evaluated by western blot analysis. (**B**) The inhibitory effect of ECN on phosphorylation of Janus kinase (JAKs) and protein tyrosine phosphatases (PTPs) was assessed by western blot analysis.

We examined the level of phosphorylation EGFR and JAKs proteins in MDA-MB-231 cells treated with ECN to determine whether the inhibitory effect of ECN on STAT3 phosphorylation is associated with the suppression of upstream signaling pathways. As shown in Figure 4A, ECN did not reduce the protein levels of phospho-EGFR. However, ECN decreased the phosphorylation of JAK1, JAK2, and Src at the upper factor of STAT3. In addition, the effect of ECN on various protein tyrosine phosphatases (PTPs), related to regulation of the JAK and STAT3 signaling was observed. The protein expressions of PTPs, SHP-1, and SHP-2 were not affected by ECN, as shown in Figure 4B. 

### 3.4. Effect of ECN on STAT3 Downstream Signaling and Caspase-Mediated Apoptosis in MDA-MB-231 Cells

**Figure 5:**
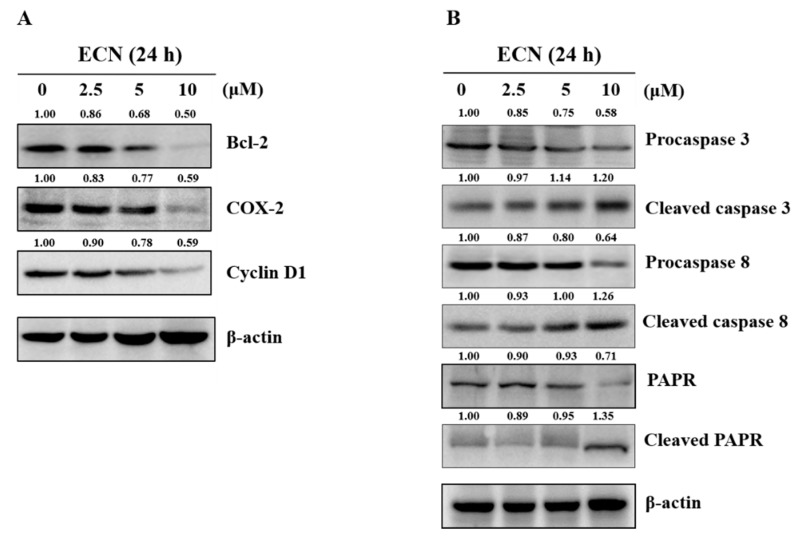
Effect of ECN on downstream signaling and caspase-mediated apoptosis in MDA-MB-231 cells. (**A**) The cells were treated with the indicated concentrations of ECN for 24 h and western blot analysis was carried out with Bcl-2, COX-2, and Cyclin D1 antibodies. (**B**) The cells were treated with the indicated concentrations of ECN for 24 h. Cell lysates were western blotted with antibodies against caspase 3, caspase 8, and PARP.

STAT3 activation has been reported to regulate the expression of various genes involved in cell proliferation, survival, angiogenesis, and cell cycle progression [41]. Therefore, the effect of ECN on STAT3 target genes in MDA-MB-231 cells was assessed. The result suggested that protein levels of Bcl-2 associated with the anti-apoptotic protein, COX-2 involved in metastasis, and cell cycle regulator Cyclin D1 were reduced by treatment of ECN, as shown in Figure 5A. In addition, proteins implicated in apoptosis were evaluated using western blot to demonstrate the anti-proliferative effect of ECN. The activation level of cleaved caspase 3, cleaved caspase 8, and cleaved PARP were increased in a dose-dependent manner after 24 h of ECN treatment, as shown in Figure 5B. 

### 3.5. Effect of ECN on the Induction of Cell Death in MDA-MB-231 Cells

**Figure 6:**
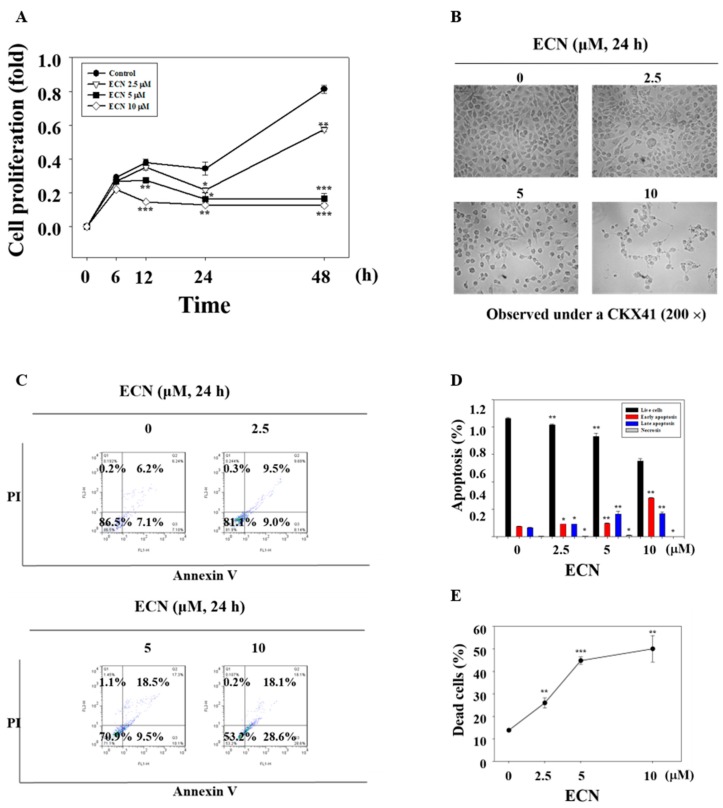
ECN induces apoptosis. (**A**) MDA-MB-231 cells were pretreated with indicated doses of ECN at different times. The cell proliferation was determined using 3-(4,5-dimethylthiazol-2-yl)-2,5-diphenyltetrazolium bromide (MTT) assay. (**B**) The cells were treated with the indicated concentrations of ECN for 24 h. The cells were washed with Dulbecco’s phosphate buffered saline (DPBS) before being fixed with 70% ethanol for 15 min and rewashed with DPBS. The cell morphology was observed under a microscope at a magnification of 200×. (**C**) The cells were treated with the indicated concentrations of ECN for 24 h and analyzed by flow cytometry assay in which 1000 events were counted per sample. The lower right quadrant represented early apoptosis and the upper right quadrant represented late apoptosis. (**D**) The percentage of live, early apoptotic, late apoptotic, and necrotic cells was calculated. (**E**) The values of dead cells included early and late apoptosis. Data were derived from three independent experiments and expressed as mean ± standard deviation. Significant difference compared with the control groups [* *P* < 0.05; ** *P* < 0.01; *** *P* < 0.001].

Along with cytotoxicity, treating the MDA-MB-231 cells with ECN inhibited cell proliferation and caused cytotoxic effect in a dose and time-dependent manner, as shown in Figure 6A. In addition, cell morphological changes were observed by microscope. When apoptosis occurs in cells, the cells undergo cell rounding, cytoplasmic condensation, and cell fragmentation, as shown in Figure 6B. For the quantitative investigation of ECN on apoptosis, MDA-MB-231 cells were incubated with ECN for 24 h and cells were analyzed by flow cytometry with Annexin V and propidium iodide (PI) staining, as shown in Figure 6C. The quantification of Annexin V binding to the cell membrane is a useful tool in detecting apoptotic cells [42]. PI is a florescent dye binding to DNA used for identifying dead cells. Comparing with the control group, ECN triggered early apoptosis in MDA-MB-231 cells in a dose-dependent manner, as shown in Figure 6D. In addition, the rates of dead cells including early and late apoptosis were increased markedly. The values of dead cell were 13.8%, 26.0%, 44.8%, and 50.1%, as shown in Figure 6E. 

### 3.6. Growth Inhibition of MDA-MB-231 Breast Cancer Xenografts by ECN in Nude Mice

**Figure 7:**
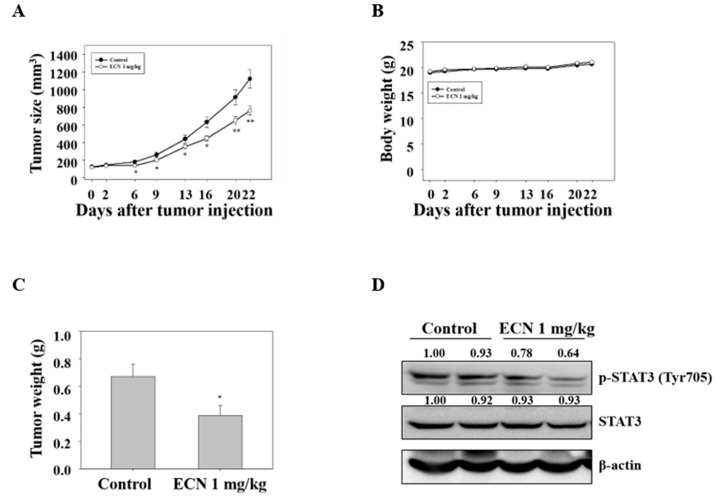
Effect of ECN on tumor growth in xenografted nude mice. (**A**) The BALB/c nude mice were injected with MDA-MB-231 cells and intraperitoneally administered with 1 mg/kg of ECN for 21 days (every 2 days). Tumor sizes were measured with a caliper every 2 days. (**B**) The body weight changes were monitored during the test period. (**C**) The tumor weights were measured. (**D**) Tumor cell lysates were western blotted with antibodies against p-STAT3 and STAT3. Data were presented as mean ± the standard error of the mean. Significant difference compared with the control groups [* *P* < 0.05; ** *P* < 0.01].

To confirm the anti-tumor effect of ECN in vivo, a xenograft assay of MDA-MB-231 cells was performed in mice (n = 10/group). BALB/c nude mice were injected with MDA-MB-231 cells and intraperitoneally administered 1 mg/kg ECN for 21 days. The average tumor size of mice treated with 1 mg/kg ECN was lower than control mice, as shown in Figure 7A. In addition, mice treated with 1 mg/kg ECN resulted in a reduction in the tumor weight (0.39 ± 0.07 g) compared with the control mice tumor weight (0.67 ± 0.08 g), as shown in Figure 7C. However, the administration of ECN did not have an effect on the overall body weight during the experimental period, as shown in Figure 7B. These results indicated that the administration of ECN inhibited the growth of MDA-MB-231 breast cancer xenograft tumors. In addition, western blot was performed using specific antibodies against p-STAT3 and STAT3 in tumor tissues. The administration of ECN resulted in a decrease in the expression of p-STAT3 consistent with the in vitro assay, as shown in Figure 7D. 

## 4. Discussion

Breast cancer is a leading cause of cancer death among females [1]. Its clinical progression is difficult to predict using current prognostic factors and its treatment is therefore not as effective as it should be [43]. TNBC, which defines the absence of staining for the estrogen receptor, progesterone receptor, and HER2, is an aggressive cancer with a poor survival rate regardless of stage [7,8,9]. However, studies have shown that patients who receive chemotherapy were healed of TNBC [44]. STAT3 is a transcription factor which has been implicated as a constitutively active oncogene in TNBC. Studies have revealed that STAT3 has a key role in regulating invasion and metastasis [29]. Therefore, therapies targeting STAT3 are important and required for preventing TNBC metastasis [45,46,47,48,49]. In this study, the anticancer effect of sesquiterpenoid from Farfarae Flos in TNBC cells, MDA-MB-231 cells, was invested.

Activity-guided fractionation and purification processes were employed to identify the anticancer compound from Farfarae Flos. Dried and chopped Farfarae Flos was extracted with 55% acetonitrile and separated with various chromatographic techniques. The extract was fractionated into sesquiterpenoids fractions by CCC and separated into 18 fractions using preparative-HPLC. Fraction P18, which showed potent anticancer activity, was purified and isolated using preparative-HPLC. An isolated compound, P18-2, was identified by comparing MS and NMR spectra data with literature to be 7β-(3-ethyl-*cis*-crotonoyloxy)-1α-(2-methylbutyryloxy)-3,14-dehydro-*Z*-notonipetranone (ECN).

ECN is an active sesquiterpenoid constituent of Farfarae Flos. Several studies revealed the biological activities of ECN. It has been reported that ECN exhibited neuroprotective effects against oxidative stress induced cell damage and dopaminergic neurodegeneration in mice [50]. ECN also demonstrated a significant inhibitory activity on NO production [51]. In addition, it inhibited against microsomal DGA1 derived from rat liver and human hepatocellular carcinoma HepG2 cells [52]. Numerous studies have shown inflammatory effects on ECN, however, there are no reports that ECN induced cell death and apoptosis in breast cancer cell. Thus, we investigated the effect of ECN on cell death in MDA-MB-231 cells both in vitro and in vivo.

The results indicated that ECN suppressed STAT3 tyrosine phosphorylation. ECN blocked nuclear translocation of STAT3, inducing cell death. Several upstream kinases, such as JAKs, Src, and EGFR are known to regulate STAT3 activation [53]. Thus, the inhibitory effect of ECN on activation of EGFR, JAK1, JAK2, and Src was investigated. ECN did not affect the protein levels of EGFR. However, phosphorylation of JAK1, JAK2, and Src proteins were affected by ECN. Moreover, ECN decreased expression of STAT3 downstream target genes, including Bcl-2, Cyclin D1, and COX-2 [41]. These results suggested that ECN inhibited JAK phosphorylation and STAT3 activation. 

Furthermore, treatment of ECN resulted in the inhibition of cell proliferation and induction of apoptosis via cleaved caspase 3, cleaved caspase 8, and cleaved PARP activation, which means that ECN induced apoptosis in MDA-MB-231 cells through extrinsic and intrinsic apoptosis pathways. To confirm the activation of apoptosis, cell morphology observation and a flow cytometry assay were conducted. The phase of apoptosis is characterized by changes in cell morphology such as cell concentration and membrane blebbing [54]. After treatment of ECN, cells were observed as round, isolated, and membrane blebbing. In addition, the rates of dead cells including early apoptotic and late apoptotic cells were increased in flow cytometry analysis. Finally, the inhibitory effect of ECN on tumor growth in a breast cancer xenograft model was evaluated. It was shown that the treatment of ECN decreased tumor sizes and tumor weights compared with the control group and ECN inhibited STAT3 activation in tumor tissues. 

In conclusion, we separated ECN from Farfarae Flos. We demonstrated that ECN inhibited the JAK–STAT3 signaling pathway inducing apoptosis of TNBC MDA-MB-231 cells. These results suggested that ECN could be a therapeutic cancer candidate against MDA-MB-231 breast cancer cells.

## 5. Conclusions

**Figure 8:**
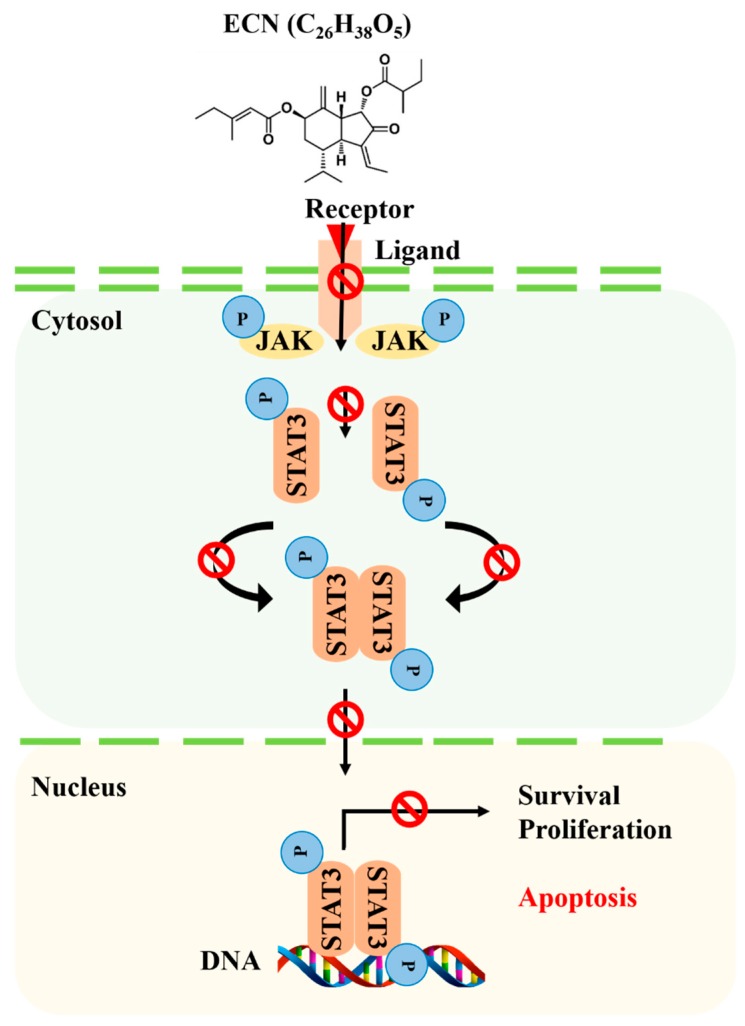
Proposed mechanism of anticancer effect of ECN by mediating STAT3 signaling.

In this study, efficient separation methods and an MTT assay were conducted to isolate an anticancer compound from Farfarae Flos. A compound—ECN—showed potent cytotoxic effect against MDA-MB-231 breast cancer cells. The study revealed that ECN inhibited JAK–STAT3 signaling and suppressed the expression of STAT3 target genes, as shown in Figure 8. In addition, ECN induced apoptosis through both extrinsic and intrinsic pathways. Cell morphology and flow cytometry analysis results suggested that ECN caused an apoptotic effect dose-dependently against MDA-MB-231 cells. Based on these results, ECN was intraperitoneally administered in mice injected with MDA-MB-231 cells. It was demonstrated that ECN suppressed activation of STAT3 and inhibited the growth of tumors. Therefore, ECN can be an effective chemotherapeutic agent for breast cancer treatment.

## Figures and Tables

**Table 1:** Inhibitory effect of fractions on the proliferation in MDA-MB-231 cells. Data represent the mean ± standard deviation from three separated experiments. Staurosporine was used as a positive control.

**Fraction**	P1	P2	P3	P4	P5	P6	P7
**IC_50_ (μg/mL)**	>30	>30	>30	>30	>30	>30	>30
**Fraction**	P8	P9	P10	P11	P12	P13	P14
**IC_50_ (μg/mL)**	>30	>30	>30	>30	>30	>30	>30
**Fraction**	P15	P16	P17	P18	PC^1^	Staurosporine	
**IC_50_ (μg/mL)**	>30	>30	>10	3.27 ± 0.18	IC_50_ (μM)	0.30 ± 0.02	

PC^1^: positive control.

**Table 2:** Inhibitory effect of P18 fraction on the proliferation in MDA-MB-231 cells. Data represent the mean ± standard deviation from three separated experiments. Fraction P18-2 was a pure compound, however fraction P18-1 and P18-3 were mixtures. Staurosporine was used as a positive control.

Fraction	IC_50_ (μg/mL)	IC_50_ (μM)
P18-1	>30	-
P18-2	-	6.85 ± 0.03
P18-3	>30	-
Staurosporine	-	0.30 ± 0.02

**Table 3:** ^1^H and ^13^C NMR assignment of P18-2 (ECN).

Position	*δ*_H_ (*J* in Hz)	*δ*_C_
1α		72.5
1β	5.52 (1H, d, 4.0)	
2		200.3
3β		139.3
4α	2.79 (1H, m)	44.9
5β	2.03 (1H, m)	41.1
6α	1.55 m (1H, m)	29.9
6β	2.31 m (1H, m)	
7α	5.51 (1H, d, 3.3)	73.5
8		141
9β	2.70 (1H, m)	46
10	6.17 (1H, s)	112.8
10’	4.81 (1H, s)	
11	2.02 (1H, m)	27.7
12	0.98 (3H, d, 6.2)	27
13	0.88 (3H, d, 7.2)	15.7
14	6.39 (1H, q, 7.2)	137
15	2.18 (3H, d, 7.2)	15.3
1′		166.1
2′	5.63 (1H, s)	114.7
3′		162.3
4′	2.18 (2H, m)	34
5′	1.07 (3H, t, 7.2)	12.1
6′	2.15 (3H, s)	19.1
1″		175.7
2″	2.40 (1H, m)	40.6
3″	1.65 (2H, m)	11.6
4″	0.90 (3H, t, 7.2)	16.7
5″	1.13 (3H, d, 7.2)	21.5

*δ* in ppm, *J* in Hz, 600 and 150 MHz in CDCl_3_.

## Supplementary Materials

The Supplementary Materials are available online.
