# Supplementary material for: A Sesquiterpenoid from Farfarae Flos Induces Apoptosis of MDA-MB-231 Human Breast Cancer Cells through Inhibition of JAK–STAT3 Signaling
Source: Biomolecules. 2019 Jul 13;9(7):278. doi: 10.3390/biom9070278 (PMC6681226; doi:10.3390/biom9070278)
Supplement: Supplementary file 1 [file biomolecules-09-00278-s001.pdf]

Article

A Sesquiterpenoid from Farfarae Flos Induces Apoptosis of MDA-MB-231 Human Breast Cancer Cells through Inhibition of JAK–STAT3 Signaling

Hyeri Jang, Hyejin Ko, Kwangho Song and Yeong Shik Kim*

Natural Products Research Institute, College of Pharmacy, Seoul National University, 1 Gwanak-ro, Gwanak-gu, Seoul 08826, Korea

* Correspondence: kims@snu.ac.kr; Tel.: +82-2-880-2479

Appendix A. Supplementary data

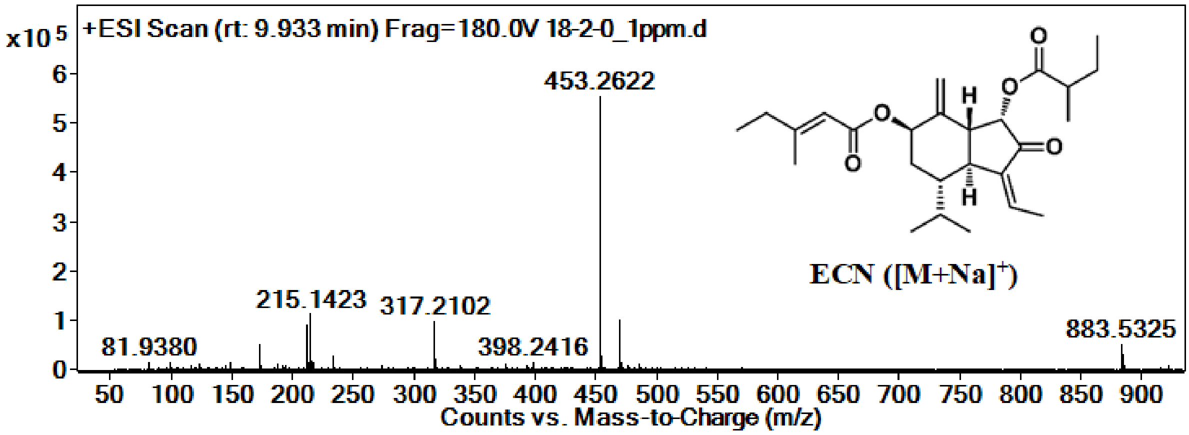

Figure S1. The representative MS spectrum of ECN at *m/z* 453.2662 $[M+Na]^{+}$.
